# Materialism and envy as mediators between upward social comparison on social network sites and online compulsive buying among college students

**DOI:** 10.3389/fpsyg.2023.1085344

**Published:** 2023-03-09

**Authors:** Yi Ling, Bin Gao, Bo Jiang, Changqing Fu, Juan Zhang

**Affiliations:** ^1^College of Education, Soochow University, Suzhou, China; ^2^College of Education, Shanghai Normal University, Shanghai, China; ^3^College of Humanities and Social Sciences, Hubei University of Medicine, Shiyan, China

**Keywords:** upward social comparison on SNS, materialism, envy, online compulsive buying, college students

## Abstract

Upward social comparison on Social Network Sites (SNS) might be positively related to online compulsive buying; however, there is little understanding of the mechanism of this relationship. In this study, we explored the effect of upward social comparison on SNS on online compulsive buying, and whether this effect is mediated by materialism and envy. A total of 568 Chinese undergraduates (mean age = 19.58 years, *SD* = 1.43) were recruited to complete a survey that included Upward social comparison on SNS Scale, Materialism Scale, Envy Scale, and Online compulsive buying Scale. The results revealed that upward social comparison was positively linked to online compulsive buying. Additionally, materialism and envy completely mediated this relationship. Our findings suggest that upward social comparison has a positive influence on college students’ online compulsive buying and that this influence is formed through a combination of cognitive factors (materialism) and affective factors (envy). This discovery not only clarifies the underlying mechanism but also proposes a potential way of alleviating online compulsive buying.

## 1. Introduction

The popularity of the Internet has changed people’s lifestyles, especially regarding shopping (Chan and Prendergast, 2007; Gosling and Mason, 2015). In December 2020, the China Internet Network Information Center announced that the population of online buyers in China had reached 842 million, with college students having the greatest potential for online buying (CNNIC, 2022). Zeng and Chen (2015) have found that the total prevalence rate of online compulsive buying among Chinese college students was 8.5%. Furthermore, a recent study shows that online compulsive shopping is on the rise in the context of COVID-19 (Maraz and Yi, 2022). The ensuing negative press is commonplace, attracting widespread attention from society and academia. Online compulsive buying, defined as a person’s inability to restrain themselves from making excessive online purchases, is a common malpractice in the information age (He et al., 2018) and may cause more harm than traditional offline compulsive buying. To understand compulsive buying, the existing differences between impulse buying and compulsive buying needs to be made. First, impulsive buying refers to the spontaneous or sudden desire to buy something, which is considered emotional, reactive, and “prone to occur with diminished regard” for the consequences (Rook, 1987). Compulsive buying is defined as “repetitive and seemingly purposeful” purchasing behaviors that cannot be controlled, are excessive, time consuming, and/or patterned in nature (Sneath et al., 2008). Distinct from impulse buying behavior, compulsive buying involves “an inability to control the impulse” (Faber et al., 1995) and “leads to extreme negative situations” (Ridgway et al., 2006). In addition, impulsive and compulsive buying have different stimuli for initiating the buying behavior. Impulsive buying are often motivated by external triggers (e.g., product promotions), so consumers are motivated to buy based on their preference for the product. Compulsive buying, however, like compulsive symptoms in general, are motivated by an internal trigger (e.g., anxiety) that stimulates the individual to spend money as an escape, and the consumer does not care about the product purchased but only about the feeling at the time of purchase. This is because the psychological experience generated by the purchase activity can help consumers escape from the stress of real life (DeSarbo and Edwards, 1996; Cai et al., 2004). Previous research has demonstrated that online compulsive buying can have certain undesirable outcomes, including emotional issues (post-purchase regret, guilt, depression, and anxiety; Gallagher et al., 2017), interpersonal conflict (poor family ties) (Trotzke et al., 2017), financial stress (severe personal debt; She et al., 2021), and low life satisfaction (Wang, 2019; Olsen et al., 2022). In addition, college students are still in the developmental stage from psychological immaturity to gradual maturity and often lack independent and clear self-awareness; moreover, their consumption behavior patterns are highly susceptible to others’ influence (Cheng, 2015). Thus, exploring the internal mechanism of online compulsive buying among Chinese college students, with extremely high rates of online buying and cell phone payments, has great theoretical value and viable importance.

Recent studies have provided evidence that online compulsive buying is influenced by personal psychological factors, such as high impulsivity, high anxiety sensitivity (Brunelle and Grossman, 2022), and high perceived stress (Zheng et al., 2020). Furthermore, it is also affected by environmental factors, such as parental materialism (Ding et al., 2019) and social comparison (Islam et al., 2018). Upward social comparison on Social Networking Services (SNS) is very common among college students. Previous research has indicated that there is a close association between upward social comparison and online compulsive buying (Zheng et al., 2020). However, the underlying mechanism of the link between upward social comparison on SNS and online compulsive buying (e.g., how this relationship occurs) remains unclear.

Upward social comparison means that people usually compare themselves with others more capable or who contribute more than they do (Collins, 1996). As a result of impression management strategies, users display information on SNS with an emphasis on positive characteristics and pleasurable experiences (Utz, 2015; Zhang et al., 2017). Consequently, they are frequently exposed to highly managed or even unrealistic “dynamics” on SNS and are thus more inclined to perceive others as having a better life than their own (Chou and Edge, 2012). Specifically, individuals frequently make upward social comparisons with others based on social status (Yaple and Yu, 2020), appearance (Etherson et al., 2022), fitness (Kim, 2022), weight loss (Wayment et al., 2020), and consumption (Mills et al., 2020). Upward social comparison on SNS is more prevalent and spontaneous among Chinese college students who use social networks, including WeChat, QQ, Weibo, and live-streaming platforms (Li et al., 2019).

The Person-Affect-Cognition-Execution (I-PACE) model can be used to interpret compulsive behavior disorders, such as online compulsive buying. The I-PACE model considers problematic behavior as the result of the interaction of personal predisposing variables and specific situations (Brand et al., 2019). Individuals may perceive external or internal triggers in specific situations, and these perceptions may trigger emotional and cognitive responses that increase the individual’s impulse to act in a particular way. Some studies have explored the relationship between upward social comparison on SNS and online compulsive buying (Kukar-Kinney et al., 2016). The findings imply that the likelihood of online compulsive buying increases with the level of social comparison of individuals on SNS (Zheng et al., 2020). From a theoretical perspective of I-PACE, individuals find contextual factors in SNS, such as upward social comparison messages, which may activate the cognitive responses of shoppers, reduce the individual’s self-control, and ultimately result in certain unfavorable behaviors on SNS, such as online compulsive buying (Brand et al., 2019). Furthermore, based on the three-stage self-regulatory process perspective of social cognitive theory, LaRose (2001) has argued that the online buying environment compensates for the lack of sensory experiences, social shopping stimuli, and immediate gratification of consumers. The online environment contains a large amount of upward social comparison information that disrupts consumers’ self-observation and self-judgment, influencing their self-reactions. Social comparison theory claims that comparison with other people is one of the basic psychological needs of individuals (Festinger, 1954). When people browse online consumer information, they can see likes and comments, which may potentially influence consumers’ self-judgment of shopping information (Li and Xue, 2022). Furthermore, the Select-Priming Model suggests that upward comparison is self-threatening and reduces subjective well-being and self-esteem (Wheeler and Miyake, 1992; Van de Ven et al., 2015), which may contribute to negative self-judgment; negative self-judgment is heightened by upward social comparison information and stimulating non-constrained buying behavior. Therefore, this study proposes hypothesis

*H1*: Upward social comparison on SNS is positively associated with online compulsive buying among college students.

Materialism is the belief that the central goals in life are the acquisition of belongings, financial success, attainment of prestige, and having an appropriate image (Kasser et al., 2004). Based on the I-PACE model, materialism represents a cognitive style that triggers compulsive online buying (Brand et al., 2019). People who hold materialistic values seek material satisfaction, and compulsive buying can satisfy their psychological needs (Richins, 2004; Ding et al., 2019). Prior studies have found that those who have a high propensity for social comparison are more susceptible to its negative effects (Hu and Guo, 2021); further, they believe that the more material wealth they have, the higher their social status (Richins, 1995; Ren et al., 2017; Xie et al., 2017), which in turn leads to increased materialism. Of course, not all upward social comparisons activate materialism. Following previous empirical evidence, upward social comparison is one of the important causes of materialism (Hu and Liu, 2020). In the Chinese cultural context, the reform and opening-up policy has been carried out for more than 40 years, and although the economy has been able to develop rapidly, it has resulted in a large gap between the rich and the poor (Xie and Zhou, 2014). As a result, people are more likely to view material wealth as a criterion for their success and use the amount of material wealth to make upward social comparisons with others to highlight their social status and identity (Zhao et al., 2022). Thus, individuals with high social comparison tendencies may be more likely to desire more material possessions and have higher levels of materialism (Chan and Prendergast, 2007). Individuals with materialistic values develop a constant need for material satisfaction, which leads to online compulsive buying. Accordingly, this study proposes hypothesis

*H2*: Materialism mediates the relation between upward social comparison and online compulsive buying.

Envy refers to an uncomfortable and painful emotion characterized by feelings of inadequacy, animosity, and resentment produced by being aware that another individual or group enjoys a coveted possession (Smith and Kim, 2007). According to the I-PACE model, upward social comparisons may elicit emotional responses (e.g., envy; Meier et al., 2020) that can motivate people to take action to obtain what they want (Crusius and Mussweiler, 2012; Lin, 2018). Hence, individuals are more likely to involve themselves in problematic behaviors, such as online compulsive buying. Furthermore, previous research has revealed that upward social comparisons can cause individuals to feel strongly socially isolated (Shensa et al., 2016), which can lead to adverse emotions. Positive messages on SNS can potentially trigger envy in individuals (Chou and Edge, 2012; Zhang et al., 2016). According to the generalized tension theory, the tension and stress experienced by individuals may lead to some nonadaptive behaviors (Agnew, 1985). Therefore, envy may be the cause of dysfunctional buying behavior, such as online compulsive buying. Thus, this study proposes hypothesis

*H3*: Envy mediates the relationship between upward and online compulsive buying.

A previous empirical study has revealed that materialism is one of the key elements that influence envy (Zheng et al., 2018). People with high materialism tend not to be easily satisfied with the status quo and are possessive and envious of others (Richins and Dawson, 1992). Moreover, those with high materialism are prone to deviations in self-understanding and self-appraisal, making them vulnerable to intense envy (Wang et al., 2017; Wang, 2021). Consequently, this study proposes hypothesis

*H4*: Materialism and envy would sequentially mediate the relationship between upward social comparison and online compulsive buying.

**Figure 1:**
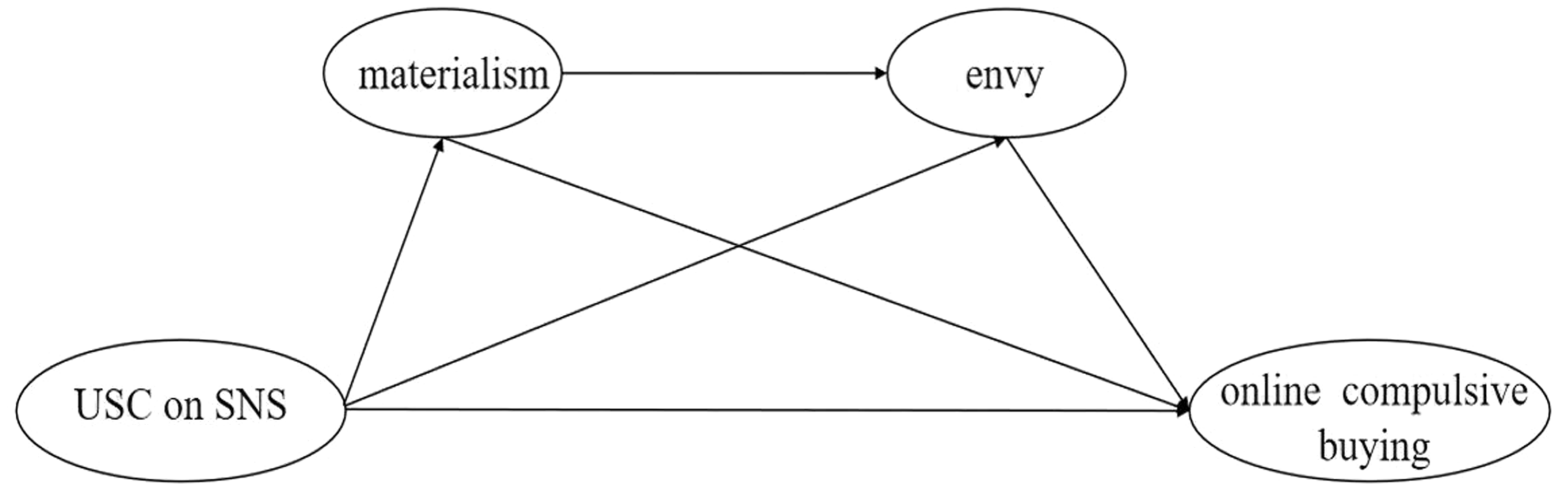
The hypothesized model.

Overall, we build on the I-PACE model to propose the hypothesized model (see Figure 1). This model explains how online compulsive buying on SNS is affected by upward social comparison, materialism, and envy. This research offers an in-depth investigation of the mechanisms between upward social comparison and online compulsive buying, which can not only expand the study of the factors influencing compulsive buying at the theoretical level but also provide guiding suggestions for preventing and intervening in college students’ online compulsive buying at the practical level.

## 2. Participants and measurements

### 2.1. Participants and data collection

The data in this study were collected during the 2021 Covid-19 period from September to November of the 2021 through Wenjuanxing (a popular online survey platform in China). We obtained valid data from 568 undergraduates from two central universities in China by clustering convenience sampling. The subjects ranged in age from 16 to 27; the mean age was 19.58 (SD = 1.43). The participants consisted of 248 (43.7%) freshmen, 117 (20.6%) sophomores, and 203 (35.7%) Junior students. Of the participants, 339 (59.7%) were males and 229 (40.3%) were females. We calculated the post-hoc statistical test power for this study using G*Power3.1 (Faul et al., 2007) software. A post-hoc power of 0.86 was achieved *via* the calculation of G-Power [Input parameters: Effect size = 0.02(consulting the main effects in Liu et al., 2019); *α* err prob. = 0.05].

### 2.2. Measurements

#### 2.2.1. Upward social comparison on SNS

The subscale from the Iowa-Netherlands Comparison Orientation Scale’s Chinese translation was used to explore upward social comparison on SNS (Bai et al., 2013), which was originally developed by Gibbons and Buunk (1999). This scale’s validity and reliability have been demonstrated among Chinese undergraduates (Liu et al., 2019). The scale is one-dimensional and has 6 items. Subjects gave their opinions on a 5-point Likert scale with values ranging from 1(Completely disagree) to 5(Completely agree). Higher scores correspond to a higher degree of USC. In the present study, Cronbach’s α for the scale was 0.92.

#### 2.2.2. Materialism

The Material Value Scale’s Chinese translation (Li and Guo, 2009) was used to assess materialism. Which has 13 items (e.g., “I do not value material things as much as most people I know”) and is divided into three dimensions: possession-defined success (five items), acquisition centrality (five items), acquisition as the pursuit of happiness (three items). Subjects gave their opinions on a 5-point Likert scale with values ranging from 1 (strongly disagree) to 5 (strongly agree). Responses to the 13 items were averaged to yield a total score. Higher scores correspond to a higher degree of materialism. In the present study, Cronbach’s α for the scale was 0.76.

#### 2.2.3. Envy

The Chinese version of the Envy Scale was applied to assess envy (Smith et al., 1999; Guo et al., 2013). The scale has a good level of reliability and validity, according to earlier studies utilizing Chinese undergraduates as the subjects (Guo et al., 2013). There are 8 items on the scale (e.g., “It depresses me to see how easy it is for others to succeed”) and is a one-dimensional questionnaire, subjects gave their opinion on a 5-point Likert scale with values ranging from 1 (Completely disagree) to 5 (Completely agree). Higher scores correspond to a higher degree of envy. In the present study, Cronbach’s α for the scale was 0.92.

#### 2.2.4. Online compulsive buying

We adopted the Chinese version of Online Compulsive Buying Scale (Zeng, 2014), which includes 13 items (e.g., “I often buy something online without planning”) and is divided into three dimensions: online buying compulsive impulse (four items), online buying compulsive behavior (four items), and negative emotions after online buying (five items). Subjects gave their opinion on a 5-point Likert scale with values ranging from 1 (strongly disagree) to 5 (strongly agree). Higher scores correspond to a higher degree of OCB. In the present study, Cronbach’s α for this scale was 0.95.

### 2.3. Control variables

Prior studies have suggested age and gender was linked to college students’ online compulsive buying (He et al., 2018; Zheng et al., 2020). Thus, in this study, gender and age were considered as control variables to reduce their confounding effects on the outcome variable.

### 2.4. Procedure

The university ethics committee of the first author approved the current work. Participants filled out questionnaires in a classroom and were given instructions on how to fill in the questionnaire, with their anonymity emphasized before data collection. Any participant might leave the study whenever they wanted.

### 2.5. Data analyzes

All statistical analyzes were performed with the SPSS 23.0 and Mplus 8.3 software. First, descriptive statistics and correlation analyzes were performed using SPSS. Descriptive analyzes were reported with a mean (*M*) and standard deviation (SD). Then, structural equation modeling (SEM) in Mplus 8.3 was used to investigate the mediating effects. The statistical significance of the pathways and indirect effects in each model was examined using a bootstrapping approach. In preprocessing the data, the full information maximum likelihood method (FIML) estimation was used to compensate for any missing data. To determine whether the model successfully matches the data, several fit indices were considered: *χ*^2^/df, CFI, TLI, RMSEA, and SRMR.

The current study used Harman’s single-factor analysis in accordance with the suggestions made by Podsakoff et al. (2003) to control the common method bias. Results found that the total variance extracted by the first factor was 30.81%, which was less than the recommended threshold of 50%.

## 3. Results

### 3.1. Descriptive statistics

Table 1 presents the descriptive statistics and correlation matrix. Higher levels of USC were positively associated with greater materialism, greater OCB, and greater envy. Similarly, materialism was positively linked with OCB and envy. OCB was positively linked with envy.

**Table 1:** Descriptive statistics and correlations between variables.

Variables	*M*	SD	1	2	3	4
1. USC on SNS	2.71	0.97	–			
2. Materialism	2.92	0.54	0.37^∗∗^	–		
3. Envy	2.18	0.89	0.47^∗∗^	0.32^∗∗^	–	
4. OCB	2.06	0.87	0.29^∗∗^	0.41^∗∗^	0.43^∗∗^	–

*N* = 568. ^**^p < 0.01. OCB, Online compulsive buying; USC on SNS, Upward social comparison on SNS.

### 3.2. The multiple mediation model

We used Mplus8.3 software to conduct confirmatory factor analysis (CFA) to evaluate the overall fit of the measurement model. As shown in Table 2, the goodness-of-fit indices for all scales have acceptable construct validity (CFI, TLI > 0.90, SRMR, RMSEA <0.08; Marsh et al., 2004). Since the Upward Social Comparison Questionnaire, the Gratitude Scale, and the Envy Scale are single-dimensional scales. Following the suggestions of Wu and Wen (2011), we used item parceling strategies in the structural equation modeling, which the items of each questionnaire were integrated into three latent variables by calculating the mean indices.

**Table 2:** The goodness of fit of the measurement model.

	*χ*^2^/df	CFI	TLI	SRMR	RMSEA
1.Upward social comparison on scale	5.42	0.99	0.99	0.011	0.042
2.Material value scale	3.30	0.93	0.91	0.052	0.064
3.Envy scale	5.42	0.98	0.96	0.031	0.069
4.Online compulsive buying scale	6.67	0.95	0.93	0.036	0.073

**Figure 2:**
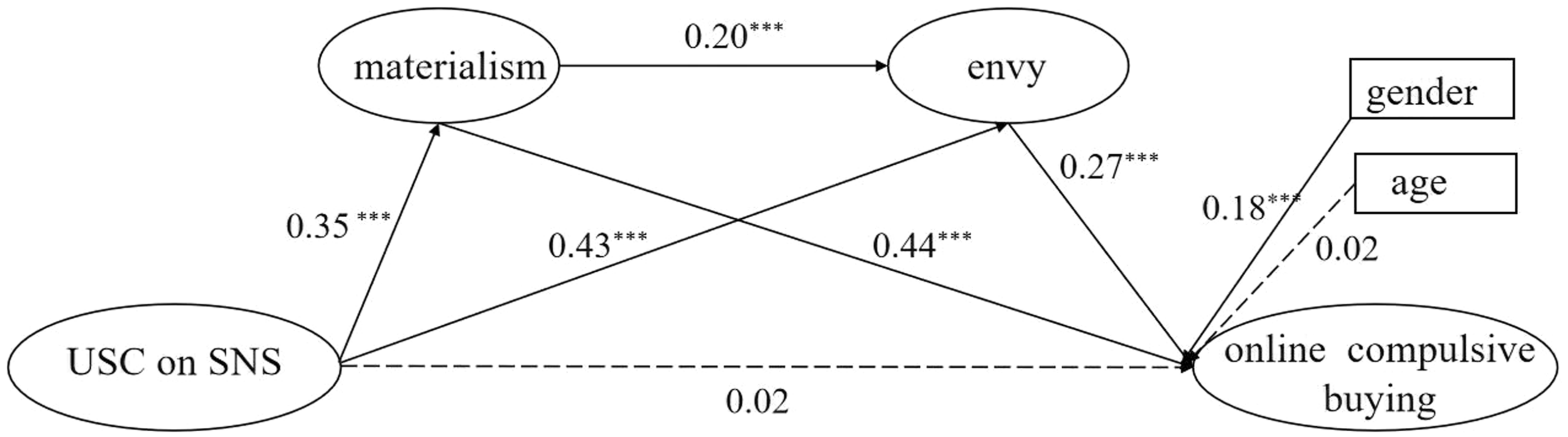
The multiple mediation model.

To test our hypotheses, Mplus 8.3 was adopted for testing the structural equation model (SEM). First, we established a model including multiple mediators based on the theoretical framework. Further, gender and grade were introduced into this model. The fit index of this model is: *χ*^2^/df = 2.51, CFI = 0.97, TLI = 0.96, RMSEA = 0.052, SRMR = 0.048.These data indicate that each fit index is good and the model is acceptable. The path coefficients are shown in Figure 2.

Second, the bias-corrected percentile Bootstrap method (1,000 random samples) was used to analyze multiple mediation effects. The direct effects and indirect effects are shown in Table 3. The results indicate that the 95% confidence intervals for all paths except the direct path do not include 0, validating the multiple mediating roles of materialism and envy in USC and OCB. Overall, the multiple mediating effects made up 93.54% of the total effect. All findings supported our given Hypotheses 1–4.

**Table 3:** Testing the pathways of the multiple mediation model.

Effect	*β*	SE	Bootstrap 95%CI	
			Lower 2.5%	Upper 2.5%
Direct effects				
Upward social comparison→materialism	0.35^***^	0.07	0.25	0.44
Upward social comparison→envy	0.43^***^	0.05	0.32	0.52
Materialism→envy	0.20^***^	0.05	0.10	0.29
Upward social comparison→online compulsive buying	0.02	0.05	−0.06	0.10
Materialism→online compulsive buying	0.44^***^	0.13	0.35	0.52
Envy→online compulsive buying	0.27^***^	0.05	0.14	0.35
Indirect effects				
Upward social comparison→materialism	0.15^***^	0.03	0.09	0.21
→Online compulsive buying				
Upward social comparison→envy	0.12^***^	0.03	0.06	0.16
→Online compulsive buying				
Upward social comparison→Materialism	0.02^***^	0.01	0.01	0.03
→Envy→online compulsive buying				

*N* = 568. ^***^p < 0.001. Bootstrap sample size = 1000.

## 4. Discussion

By reviewing the previous literature, we have found that this is the first study that attempts to identify the fundamental mechanism between upward social comparison and online compulsive buying. The results offer an insight into the psychological mechanisms of materialism and envy as mediators of the influence of upward social comparison on online compulsive buying. This section further discusses the main outcomes.

Previous research has demonstrated that upward social comparison may influence materialism (Ren et al., 2017; Hu and Liu, 2020) as well as inappropriate buying behavior. This result supports the I-PACE model (Brand et al., 2019), which states that upward social comparison information might cause negative cognitive reactions and that individuals may want to narrow the gap with others by accumulating more material wealth to achieve their ideal self. Therefore, materialistic individuals will find self-worth and meaning through material satisfaction (online compulsive buying; Goldberg et al., 2003; Ku et al., 2014; Jiang et al., 2016). In addition, online buying facilitates the promotion of obsessive materialism owing to its fashionable, practical, and preferential qualities (Ding et al., 2019). Empirical studies have shown that materialism effectively predicts online compulsive shopping among college students (Li et al., 2016). Therefore, materialism is a strong predictor of college students’ online compulsive buying (Li et al., 2016). Specifically, college students are more prone to use SNS to compare themselves to those in higher socioeconomic classes, thus making them more susceptible to negative impacts and having a greater propensity to exhibit compulsive buying behavior (Shaw et al., 2015).

As anticipated, we discovered that envy acts as a mediator between upward social comparison and online compulsive buying. This result is in line with the I-PACE model (Brand et al., 2019), which suggests that individuals with high levels of upward social comparison possess more envy, which increases the likelihood of their engagement in compulsive buying. Additionally, social comparison theory claims that individual envy often arises from social comparisons (Ding et al., 2017). This could be because upward social comparison frequently indicates that an individual has an unfulfilled goal, and when the interests of others are unchangeable or uncontrollable, feelings of hostility arise, leading to envy (Testa and Major, 1990). Consequently, it seems sensible to argue that envy is a normal, automatic emotional reaction to unflattering upward social comparisons. Crusius and Mussweiler (2012) have argued that envy is incited in an individual when others have what they do not have; this frustration and hostility is socially unacceptable and therefore often repressed or controlled. Once self-control is diminished, the envy-generated impulse to try to own others’ goods may become stronger, resulting in a greater willingness to buy things (Wu and Chang, 2013). Therefore, envy is a significant predictor of online compulsive buying among college students.

Furthermore, the association between upward social comparison and compulsive buying was also found to be mediated by materialism and envy, both simultaneously and sequentially. Our results are in accordance with the I-PACE model (Brand et al., 2019), in which materialism and envy can be viewed as a cognitive and affective response, respectively, both increasing the propensity to engage in online compulsive buying. Overall, the sequential mediating effects of materialism and envy link cognitive and affective factors, suggesting that college students with higher levels of social upward comparison have a stronger pursuit of material things (Wang et al., 2017; Sun and Liu, 2020). Meanwhile, materialism impairs individuals’ psychological needs satisfaction and increases envy, which in turn motivates individuals to adopt compulsive buying behaviors to satisfy their need for psychological balance.

Currently, few studies have explored the direct relationship between downward comparison and compulsive buying, but we suppose that downward comparison may lead to lower compulsive buying. Previous studies have shown that downward comparison can enhance individual self-concept (eg., self-perceived competence) and life satisfaction (Pohlmann and Möller, 2009; Huang, 2014). It is worth noting that consumers are less likely to spend money on products after making downward comparisons (Zheng et al., 2018).

Despite the meaningful results found in this study, there are still some limitations. First, the data came from subjects’ self-reports, which could lead to a social approval effect, lowering the accuracy of the findings. A multi-subject reporting method should be considered in the future to fill this gap. Second, the cross-sectional study was unable to reveal causal relationships between variables, and future longitudinal studies with experimental designs are needed to confirm relevant findings. Furthermore, this study explored the mediating role of materialism and envy, and future research could introduce some other variables (e.g., depression) for in-depth study, there are relevant empirical studies showing that upward social comparison on social networking sites is positively associated with depression (Tian et al., 2020), and previous clinical studies found that online compulsive buying is mainly driven by depression (Müller et al., 2014). Also, some moderating variables (e.g., self-control) could be included to reveal the boundary of influence of upward social comparison and online compulsive buying. Again, all data in this study came from the Chinese college student population, and previous studies have found that as China is a country that promotes a collectivist culture, people are more concerned with how they are evaluated by others and are more likely to make social comparisons (Chi, 2020). Therefore, future cross-cultural studies can be conducted to improve the generalizability of the findings. Finally, the reliability of the materialism questionnaire in this study was low, which limited the reliability of the results to a certain extent; therefore, more studies are needed to test the results of this study.

The findings of this study have important implications for guiding college students to reduce the negative effects of upward social comparison on online compulsive buying. First, reducing the level of upward social comparison can help reduce online compulsive buying; we suggest that college students should try to make fewer upward social comparisons with others on SNS, focus more on their own merits, and learn to be content. Second, to reduce the possibility of compulsive buying, college students need to know how materialistic beliefs affect their cognition, emotions, and behaviors, making efforts to reduce the negative effects of materialism. Third, reducing envy may help reduce online compulsive buying. Schools and relevant authorities can provide cognitive-behavioral training to students in an attempt to transform malicious envy into benign envy (Liu et al., 2019).

## Data availability statement

The original contributions presented in the study are included in the article/Supplementary material, further inquiries can be directed to the corresponding author.

## Ethics statement

The studies involving human participants were reviewed and approved by Ethics Committee, Department of Psychology, Soochow University. The patients/participants provided their written informed consent to participate in this study.

## Author contributions

YL was responsible for the literature search and literature review of the paper in the early stages and was responsible for the writing of the paper (introduction and discussion) in the later stages. BG was responsible for defining the direction and framework of the study in the early stages and was involved in the writing of the paper (results section) in the later stages. BJ supervised the study throughout and provided guidance on the research methods. CF and JZ were responsible for data collection and entry in the early stages. All authors contributed to the article and approved the submitted version.

## Funding

This work was supported by grants from the National Natural Science Fund Committee, general Projects (18YJA860006).

## Conflict of interest

The authors declare that the research was conducted in the absence of any commercial or financial relationships that could be construed as a potential conflict of interest.

## Publisher’s note

All claims expressed in this article are solely those of the authors and do not necessarily represent those of their affiliated organizations, or those of the publisher, the editors and the reviewers. Any product that may be evaluated in this article, or claim that may be made by its manufacturer, is not guaranteed or endorsed by the publisher.
