# Supplementary material for: Materialism and envy as mediators between upward social comparison on social network sites and online compulsive buying among college students
Source: Front Psychol. 2023 Mar 9;14:1085344. doi: 10.3389/fpsyg.2023.1085344 (PMC10034084; doi:10.3389/fpsyg.2023.1085344)
Supplement: Supplementary file 1 [file Table_1.DOCX]

| **construct** |  | **items** | **source** |  |
| --- | --- | --- | --- | --- |
| upward social comparison on SNS(USC) | USC1 | On social network sites, I always like to compare myself with others who are better off me | Bai et al.(2013) |  |
|  | USC2 | On social network sites, I often compare myself with others who perform better than me. |  |  |
|  | USC3 | On social network sites, I always pay a lot of attention to how I do things compared with how others are doing better than me. |  |  |
|  | USC4 | On social network sites, I often compare how I am doing socially with who are better at social networking sites |  |  |
|  | USC5 | On social network sites, I am the type of person who compares often with superiors |  |  |
|  | USC6 | On social network sites, I often compare myself with superiors with respect to what I have accomplished in life |  |  |
| MaterialismScale (MS) | MS1 | I admire people who own expensive homes, cars, and clothes | Li and Guo(2009) |  |
|  | MS2 | I usually buy only the things I need |  |  |
|  | MS3 | I don't pay much attention to the material objects other people own |  |  |
|  | MS4 | Some of the most important achievements in life include acquiring material possessions |  |  |
|  | MS5 | I try to keep my life simple, as far as possessions are concerned |  |  |
|  | MS6 | I don't place much emphasis on the amount of material objects people own as a sign of success |  |  |
|  | MS7 | My life would be better if I owned certain things I don't have. |  |  |
|  | MS8 | The things I own say a lot about how well I'm doing in life |  |  |
|  | MS9 | I enjoy spending money on things that aren't practical |  |  |
|  | MS10 | I wouldn't be any happier if I owned nicer things |  |  |
|  | MS11 | Buying things gives me a lot of pleasure |  |  |
|  | MS12 | I'd be happier if I could afford to buy more things |  |  |
|  | MS13 | I like a lot of luxury in my life |  |  |
| Envy scale(ES) | ES1 | I feel envy every day | Guo et al.( 2013) |  |
|  | ES2 | The bitter truth is that I generally feel inferior to others |  |  |
|  | ES3 | Feelings of envy constantly torment me |  |  |
|  | ES4 | It is so frustrating to see some people succeed so easily |  |  |
|  | ES5 | No matter what I do, envy always plagues me |  |  |
|  | ES6 | I am troubled by feelings of inadequacy |  |  |
|  | ES7 | It somehow doesn’t seem fair that some people seem to have all the talent |  |  |
|  | ES8 | Frankly, the success of my neighbors makes me resent them |  |  |
| Online compulsive buying(OCB) | OCB1 | I often inexplicably buy things online | Zeng (2014) |  |
|  | OCB2 | Even though I'm busy, I can't help but want to shop online |  |  |
|  | OCB3 | I often buy things online without planning |  |  |
|  | OCB4 | I often feel self-conscious after a continuous online shopping frenzy |  |  |
|  | OCB5 | Even though I have no money, I still can't help but want to buy online |  |  |
|  | OCB6 | I often buy things online, even if I can't afford them at all |  |  |
|  | OCB7 | I often feel ashamed after a continuous online shopping frenzy |  |  |
|  | OCB8 | Even though I don't need anything, I can't help but want to shop online |  |  |
|  | OCB9 | I often buy things online that I don't need or have no use for |  |  |
|  | OCB10 | I often feel nervous or anxious after a continuous online shopping frenzy |  |  |
|  | OCB11 | I always make a great effort to restrain myself from the strong desire to shop online |  |  |
|  | OCB12 | I often have the thought that I must not buy so many things online next time, and feel sorry for my online shopping behavior |  |  |
|  | OCB13 | I often think "I will never shop online again" and regret my online shopping behavior |  |  |
